# Venereal Prophylaxis

**Published:** 1910-06-18

**Authors:** 


### VENEREAL PROPHYLAXIS.

The problem of the prevention of venereal disease is one that has engaged the attention of the naval and the military surgeon for many years, but such attempts as have been made to institute prophylactic treatment have, for various reasons, been sporadic and fitful. That a properly supervised and regularly carried out system of prophylaxis is effectual is proved by the figures given by Passed Assistant-Surgeon W. J. Zalesky in an able article on the subject contributed to the *U.S. Naval Medical Bulletin.* Some two years ago, when stationed at New Orleans, the author, by lecturing the men and impressing on them the importance of prophylaxis, succeeded in inducing twenty-three out of eighty-eight of them to apply for prophylactic treatment. The result was satisfactory for a time, during which no new cases were met with, but when interest in the scheme evaporated the men ceased attending and a new outbreak occurred. The author then enlisted the commanding officer, and as a result an order was promulgated in which it was stated that the men would be examined at frequent intervals by the surgeon, and that any cases of venereal disease occurring in those who had not applied for prophylactic treatment would be reported to the commanding officer, whereas no reports would be entered on the sickness or disability sheets of those carrying out preventive measures. The result of this order was an immediate improvement in the health of the men; no new cases occurred for six months. The author was then appointed to the U.S.S. *Salem*, where, with the approval of the commanding officer, prophylactic measures were again instituted. The men were taken in batches and the measures explained to them. Each member of the crew was examined, and where venereal disease existed appropriate treatment was applied, with curtailment of shore leave until cured. The liberty parties were examined daily, and on returning from leave each man who had been exposed to infection was expected to apply within twelve hours for prophylactic treatment. This consists in washing the penis and soaking it for five minutes in a 1 in 1,000 solution of mercuric bichloride, followed by an injection of a 2 per cent. solution of protargol, held in the urethra for five minutes by the clock, and, lastly, an application of 30 per cent. calomel ointment. The whole treatment thus takes about seventeen minutes of the men's time. The author remarks that protargol is not entirely satisfactory, as it in some cases sets up a non-specific urethritis by irritation, a disadvantage which is shared by other salts of silver, such as the iodide and nitrate. He has used hot potassium permanganate irrigations successfully, but they are unsatisfactory in that they need to be administered by a second person, and only one patient can be treated at a time. If prophylaxis is to be effectual every man must be examined at frequent intervals, whether a liberty man or not. The card-index system, in which each man's name and details of inspection and treatment are filled in on a separate card, provides a ready method of finding out how often each patient has been seen. As the result of four and a-half months' trial of prophylactic measures only six cases of gonorrhœa were discovered, and no new cases of syphilis. Three of the former were concealed, for which the delinquents were summarily court-martialled; and of the other three one occurred in a man who broke leave and returned after eight days with the disease fully developed, two being in men who had received prophylactic treatment. The crew consisted of 350 men, and during the time under review liberty was given at seven stopping places, there being seventy-eight liberty days, during which 5,300 liberty men were examined. The author thinks that the bulk of the work of examining cases should fall on the hospital corps, who should be properly trained to that end. The medical officer's duties should be merely supervisory, as his presence on all occasions would only tend to embarrass the men. Cases of doubt only should be referred to him. The author finds that men do not object to frequent examinations, and that after the necessary stimulus has been given to obtain their interest in the scheme, they become grateful for the care taken to preserve them from venereal disease, and willingly co-operate with their medical officer.

